# Factors of physical activity among Chinese children and adolescents: a systematic review

**DOI:** 10.1186/s12966-017-0486-y

**Published:** 2017-03-21

**Authors:** Congchao Lu, Ronald P. Stolk, Pieter J. J. Sauer, Anna Sijtsma, Rikstje Wiersma, Guowei Huang, Eva Corpeleijn

**Affiliations:** 1 Department of Epidemiology (HPC FA40), University Medical Centre Groningen, PO Box 30.001, 9700 RB Groningen, The Netherlands; 2 School of Public Health, Tianjin Medical University, Tianjin, China

**Keywords:** Physical activity, Factors, Children, Adolescents, Chinese, Review

## Abstract

**Background:**

Lack of physical activity is a growing problem in China, due to the fast economic development and changing living environment over the past two decades. The aim of this review is to summarize the factors related to physical activity in Chinese children and adolescents during this distinct period of development.

**Methods:**

A systematic search was finished on Jan 10^th^, 2017, and identified 2200 hits through PubMed and Web of Science. English-language published studies were included if they reported statistical associations between factors and physical activity. Adapted criteria from the Strengthening The Reporting of OBservational studies in Epidemiology (STROBE) statement and evaluation of the quality of prognosis studies in systematic reviews (QUIPS) were used to assess the risk of bias of the included studies. Related factors that were reported in at least three studies were summarized separately for children and adolescents using a semi-quantitative method.

**Results:**

Forty two papers (published 2002–2016) were included. Most designs were cross-sectional (79%), and most studies used questionnaires to assess physical activity. Sample size was above 1000 in 18 papers (43%). Thirty seven studies (88%) showed acceptable quality by methodological quality assessment. Most studies reported a low level of physical activity. Boys were consistently more active than girls, the parental physical activity was positively associated with children and adolescents’ physical activity, children in suburban/rural regions showed less activity than in urban regions, and, specifically in adolescents, self-efficacy was positively associated with physical activity. Family socioeconomic status and parental education were not associated with physical activity in children and adolescents.

**Conclusions:**

The studies included in this review were large but mostly of low quality in terms of study design (cross-sectional) and methods (questionnaires). Parental physical activity and self-efficacy are promising targets for future physical activity promotion programmes. The low level of physical activity raises concern, especially in suburban/rural regions. Future research is required to enhance our understanding of other influences, such as the physical environment, especially in early childhood.

**Electronic supplementary material:**

The online version of this article (doi:10.1186/s12966-017-0486-y) contains supplementary material, which is available to authorized users.

## Background

Globally, many children and adolescents are relatively inactive, mostly too inactive to meet the physical activity recommendations [1, 2]. The trend of physical inactivity is increasing rapidly in most societies around the world. This fact is not only in high-income countries but also increasingly in low- and middle-income countries [3, 4], as a consequence of the fast economic development and changing living environment over the past two decades [5–7]. For example, a rapid increase of vehicle ownership in the population is likely to reduce the need for “active transport” [8], and Chinese city children especially depend on their parents for daily transportation. As elsewhere in the world, physical inactivity is acknowledged as a key factor of human health in Chinese society [9, 10].

The health benefits of physical activity for children and adolescents are well established [11, 12]. Participation in physical activity during childhood plays an integral role in adult health outcomes, such as increased bone mineral density, and, indirectly, by preventing overweight [13]. Therefore, the World Health Organization recommends that children and adolescents aged 5–17 years should accrue at least 60 min of moderate to vigorous intensity physical activity daily [14]. Data of the Chinese “2010 National Physical Fitness and Health Surveillance” showed that 77.3% (128,890 out of 166,757 participants) of students in schools failed to meet the recommendation [15]. This level of physical activity may be too low to maintain good health. Since physical activity patterns track from childhood to adolescence and adulthood [16, 17], understanding those factors that influence physical activity during early life can aid in the design of more effective interventions to stimulate physical activity later in life, or to counteract the growing trend towards inactivity. Several comprehensive reviews of correlates of children’s and adolescents’ physical activity have been published [18–20], however, none have focused on developing countries. This review will systematically review the factors related to physical activity in Chinese children and adolescents.

## Methods

This review was conducted and is reported according to the Preferred Reporting Items for Systematic Reviews and Meta-Analyses (PRISMA) guidelines [21].

### Search procedure

A systematic search for studies investigating factors influencing physical activity in children and adolescents in China was conducted using two English-language electronic databases (Web of science and PubMed). Search terms were made up of a combination of keywords: “China” OR “Chinese” AND “child*” OR “adolescen*” OR “student*” OR “youth*” AND “physical activity” OR “activity level” OR “exercise” OR “physically active” OR “motor behavio*”. The study search was carried out before Jan 10^th^, 2017. Subsequent studies were identified by screening the reference lists of papers that fulfilled the inclusion criteria.

### Inclusion and exclusion criteria

The inclusion criteria were as follows: (1) the study was published in English; (2) the study population consisted of Chinese children or adolescents living in China (age 3–18 years, or with a mean age in this range); and (3) the study reported a measurement of physical activity as the dependent outcome and examined the statistical associations with certain factors. The exclusion criteria were as follows: (1) the study population was characterized by disabilities or an illness that could lower their ability in terms of bodily movement; (2) intervention studies and studies that measured physical activity as the independent variable were not included, unless they reported associations between related factors and physical activity as the dependent outcome; (3) studies on physical inactivity and physical activity measured in a specific setting for a limited period of the day (such as during a physical education class) were excluded; (4) studies which were only published as abstract, a comment, or review were excluded, due to a lack of data for extraction, but the reference lists were checked for relevant studies.

### Search results

**Fig. 1:**
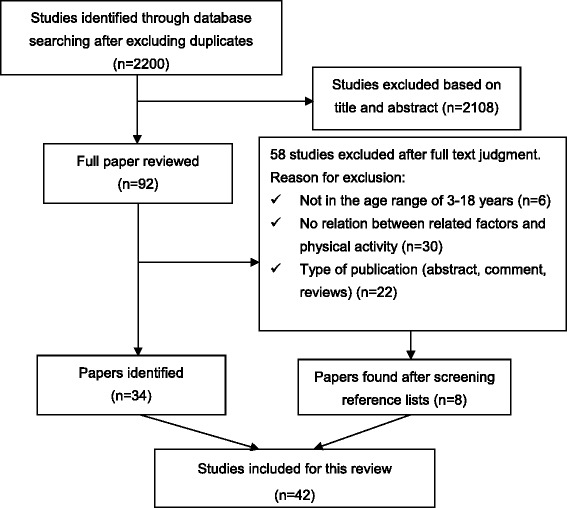
Flow chart of the literature search strategy

In total 2200 hits were identified after excluding duplicates. Ninety-two papers remained, after reading titles and abstracts for inclusion and exclusion criteria. After a review of all the papers, 42 studies were included in this review. The literature review strategy is shown in Fig. 1.

### Characteristics of the physical activity measurement

The outcomes were reported as total physical activity or leisure-time physical activity. If one study reported different intensities of physical activity, only moderate and vigorous physical activity were chosen for the summary. If both univariate and multivariate analyses were published, the adjusted multivariate results of potential correlates were selected for this review. Reported results separated by boys or girls, or different correlates for fathers or mothers, were also noted.

### Method for summarizing results

For this review, we summarized potential associations that were examined in at least three studies. For every result in each paper, it is indicated whether the identified association is positive or negative. The direction of the association is expressed by a positive “+” or negative “-” association. For the summary of each variable, the hypothesized directions of associations were based on the rules drawn up by Sallis and colleagues [18]: the result was defined as no association (coded with a “0”), if 0–33% of findings supported the association, and as an inconclusive finding (coded with a “?”), if 34–59%. If more than 59% of findings supported the association, the result was defined as a positive association (coded with a “+”) or a negative association (coded with a “-”). We presented the correlates for children and adolescents separately, because, due to differences in age, results might show differences as demonstrated in previous studies [18, 22].

### Methodological quality assessment

Criteria for assessing the quality of studies were adapted from the Strengthening The Reporting of OBservational studies in Epidemiology (STROBE) statement [23] and evaluation of the quality of prognosis studies in systematic reviews (QUIPS) [24]. Five items were considered the most important in the context of this review and were included in the checklist, including study design (met the criterion if a longitudinal design was used), study participation (met the criterion if study sample represent the population of interest in terms of key characteristics), outcome measurements (met the criterion if report a clear description of physical activity, and the instruments have an acceptable quality), related factors measurements (use validated methods and describe details of assessment), and data analysis (met the criterion if statistical tests used to assess the main outcomes appropriate, including confounding). A score was assigned to each study based on whether quality assessment items met the criterion (score = 1) or not (score = 0). Most studies used questionnaires to describe the activity level. It is known, however, that the validity of these questionnaires is limited. Therefore, in most studies we rated the validity as limited. The scores were summed and described as low quality (0–2) or acceptable quality (3–5).

Two reviewers independently screened titles and abstracts of all hits identified by the search, and the full text of all potentially eligible papers was screened for final selection by the same reviewers (CL & RW). Data extraction and the methodological quality were assessed independently by two reviewers (CL & RW); disagreement was discussed in a consensus meeting or by consulting a third reviewer (EC). The criteria for quality assessment are given in Additional file 1.

## Results

### Study characteristics

A summary of the characteristics of the 42 papers is given in Table 1. The publication period ranged from 2002 to 2010 [25–41], with 25 papers (60%) published after 2010 [42–66]. Only 9 studies were longitudinal designs. The sample sizes ranged from 50 to 29,139 participants, and 18 papers (43%) had a sample size above one thousand. Seventeen papers included young children (aged 3–12 years or mean age in the period), 20 included only adolescents (13–18 years or mean age in the period), and 5 studies included both age groups. Objective assessment of physical activity was used in nine studies, such as an accelerometer [25, 48, 56, 58–61], a pedometer [53], and heart rate monitoring [35]. The other studies used questionnaires to measure physical activity. From all the studies, 69% reported on the quality of the tools (reliability or validity) or referred to the original publication. The characteristics of the individual papers are summarized in Additional file 2.

**Table 1:** Summary of characteristics of the included papers (*N* = 42)

Characteristics of the papers	N(%)	Paper No.
Year of publication		
2002–2005	5(12)	[25–29]
2006–2010	12(29)	[30–41]
2011–2016	25(60)	[42–66]
Study design		
Longitudinal	9(21)	[29, 38, 42, 46, 51, 56–59]
Cross-sectional	33(79)	[25–28, 30–37, 39–41, 43–45, 47–50, 52–55, 60–66]
Sample size		
50 ≤ n <100	4(10)	[25, 35, 53, 59]
100 ≤ n <500	11(26)	[26, 28, 33, 39, 42, 44, 47, 50, 56, 60, 62]
500 ≤ n <1000	9(21)	[29, 30, 36, 38, 46, 54, 58, 61, 66]
1000 ≤ n <10,000	16(38)	[27, 31, 32, 34, 37, 41, 43, 45, 48, 51, 52, 55, 57, 63–65]
n >10,000	2(5)	[40, 49]
Age group		
Children (3–12 years.)	17(40)	[25, 28, 33, 36, 37, 39, 42, 44, 47, 50, 53, 56, 58–60, 64, 66]
Adolescent (13–18 years.)	20(48)	[26, 29–32, 35, 38, 40, 41, 45, 46, 49, 51, 52, 54, 55, 61–63, 65]
Both (3–18 years.)	5(12)	[27, 34, 43, 48, 57]
Method of PA measurement^a^		
Objective measurement of PA	9(21)	
Reported reliability or validity	1(2)	[25]
Mention of original reference	3(7)	[53, 60, 61]
None reported	5(12)	[35, 48, 56, 58, 59]
PA measured by questionnaires	35(83)	
Reported reliability and validity	5(12)	[28, 33, 35, 40, 56]
Reported reliability or validity	8(19)	[26, 29, 36, 39, 44, 45, 60, 63]
Mention of original reference	14(33)	[27, 31, 32, 34, 37, 41, 42, 46, 47, 50, 52, 54, 62, 65]
None reported	8(19)	[30, 38, 43, 49, 55, 57, 64, 66]

^a^Totals may add up to more than 100%, since two studies included both objective and questionnaire measurement of PA [35, 60]

### Results of the methodological quality assessment

Overall, only two studies met all five quality criteria. Eighteen studies (43%) failed to meet the criteria for participation, since the response rate was less than 80% or not clearly described. Ten studies (24%) lacked information on handling confounders, and thus failed to meet the criteria for data analysis. In sum, 37 studies (88%) showed acceptable quality, and 5 studies (12%) low quality. Low quality studies are marked in Table 2. Results of the methodological quality assessment are summarized in Additional file 3.

**Table 2:** Potential correlates of physical activity in young children and adolescents

Variables	Age group	Related to PA		Unrelated to PA	Summary	
		Paper No.	Corr^a^	Paper No.	n/N(%)	Corr^a^
Gender (male)	Children	[36], [43]^b^MVPA, [44]Lt(VPA), [48]MVPA, [52]MVPA, [53], [60]MVPA	+	[25], [27]MVPA, [28], [37]^b^MVPA	+7/11(64)	+
	Adolescents	[27]MVPA, **[29]**, [31], [32]Lt(VPA), [41]Lt, [43]^b^MVPA, [45], **[46]**Lt, [48]MVPA, [61]MVPA, [63]MVPA	+	[30]^b^, [32]Lt(MPA)	+11/13(85)	+
Mother’s PA	Children	**[42]**Lt(MVPA), [50]MVPA, **[57]**Lt	+	[34]MVPA	+3/4(75)	+
	Adolescents	**[57]**Lt, [65]MVPA	+	[34]MVPA	+2/3(67)	+
Father’s PA	Children	[34]MVPA, **[57]**Lt	+	None		
	Adolescents	[34]MVPA, **[57]**Lt, [65]MVPA	+	None	+3/3(100)	+
Self-efficacy	Children	[60]MVPA	+	None		
	Adolescents	**[29]**, **[38]**Lt, [54], [62]MVPA	+	None	+4/4(100)	+
Urbanization (urban)	Children	[33]MVPA(F), [34]MVPA, [52]MVPA	+	[36], **[42]**Lt(MVPA)	+3/5(60)	+
	Adolescents	[31], [34]MVPA [30]^b^F	+ -	[30]^b^M, [32]Lt(MPA&VPA)	+2/5(40)	?
Weight status	Children	[64]^b^	-	[47]MVPA, [48]MVPA, [53], [60]MVPA, [66]Lt	-1/6(17)	0
	Adolescents	[49], [63]MVPA	-	[31], [48]MVPA	-2/4(50)	?
SES	Children	[43]^b,c^MVPA	+	[36], **[42]**Lt(MVPA)	+1/3(33)	0
	Adolescents	[43]^b,c^MVPA, [54] [30]^b^M, [61] ^c^MVPA	+ -	[30]^b^F, [32]Lt(MPA&VPA), [63]^c^MVPA, [65]MVPA	+2/8(25)	0
Parental education	Children	None		[36], **[42]** ^d^Lt(MVPA), [43]^b^MVPA, [47]MVPA	0/4(0)	0
	Adolescents	[30]^b,d^M **[29]**	+ -	[30]^b,d^F, [31], [43]^b^MVPA,	+1/5(20)	0
Age	Children	[37]MVPA, **[42]**Lt(MVPA)	+	[28], [53], [60]MVPA	+2/5(40)	?
	Adolescents	[27]MVPA, [31], [45]	-	[30]^b^, [32]Lt(MPA&VPA), [54], [61]MVPA, [63]MVPA	-3/8(38)	?
Previous PA	Children	None				
	Adolescents	**[38]**Lt, **[46]**Lt,	+	[62]MVPA	+2/4(50)	?
		**[51]**Lt(MVPA)	-			
Intention	Children	None				
	Adolescents	[65]MVPA	+	**[38]**Lt, **[46]**Lt, [62]MVPA	+1/4(25)	0

Note. *Bold* longitudinal study, *F* female, *Lt* leisure time physical activity, *M* male, *MPA* moderate physical activity, *MVPA* moderate-to-vigorous physical activity, *PA* physical activity, *SES* socioeconomic status (household income), *Underline* objective measurement of physical activity, *VPA* vigorous physical activity

^a^ Correlates were examined in at least three studies. The result is defined as no correlation (“0”) if 0–33% of findings supported the correlation, as inconclusive (“?”), if it was 34–59%, and positive (“+”), or negative (“-”), if it was 59–100% (Sallies [18])

^b^ Low quality study

^c^ Middle level SES (family income ranging from RMB 2000 to RMB 5000 per month)

^d^ Maternal education

### Level of physical activity

**Fig. 2:**
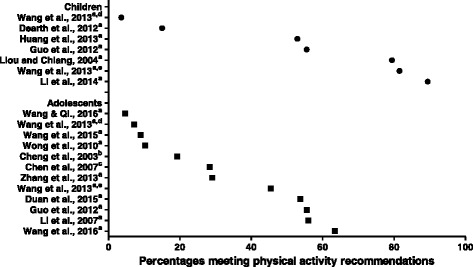
Prevalence (%) of compliance with recommendations of physical activity in 15 studies is highly variable. **a** Recommendation to participate in physical activity for at least 60 min of MVPA per day; **b** Recommendation of three or more sessions per week of activities that last 20 min. or more, and that require moderate to vigorous levels of exertion (Sallis [92]); **c** Recommendation to participate in physical activity 3 or more times a week for at least 30 min, which entails deep breathing and increased heartbeat (Taiwanese recommendations for physical activity). **d** Chinese-specific cut-off points for accelerometry used in study (Wang et al., [48]). **e** Freedson’s cut-off points for accelerometry used in study (Wang et al., [48])

The levels of physical activity described in the studies are summarized in Additional file 2. The outcomes of physical activity were varied due to different measurements. Fifteen studies provided data for children or adolescents meeting physical activity recommendations. Most studies (*N* = 13) used the same international guidelines, that is, participating in physical activity for at least 60 min of MVPA per day [14, 67, 68], and found a low level of physical activity, especially in large sample studies [40, 49]. Two studies of children and seven of adolescents reported less than 50% of participants failed to meet the recommendations. The prevalence rates of compliance to recommendations of physical activity in children range from 3.6 to 89.4%, and in adolescents range from 4.7 to 63.4%. Results of the 15 studies are presented in Fig. 2.

### Factors influencing physical activity

Potential correlates for physical activity and factors influencing the activity level are summarized in Table 2. As described before, a correlation was defined as positive if 59–100% of studies found a positive (“+”) association and as negative if 59–100% of studies found a negative (“-”) association. Eleven variables were investigated in at least three studies: gender, urbanization, maternal physical activity, paternal physical activity, weight status, socioeconomic status, parental education, self-efficacy, age, previous physical activity and intention. Gender (*N* = 20 papers), both in children and adolescents, was consistently found to have a positive association with physical activity: thus, boys were more active than girls. For maternal physical activity (*N* = 5 papers), it was shown that both children and adolescents were more active if their mother were more active. Paternal physical activity was also found (*N* = 3 papers) to have a positive association with physical activity in adolescents. Urbanization (*N* = 8 papers) yielded different results for young children and adolescents. For young children, those living in urban regions showed more physical activity than suburban/rural children. The correlates for urbanization in adolescents were inconclusive. The weight status (*N* = 9 papers) showed no association with children’s physical activity, and showed inconsistent results with adolescents’ physical activity. In both young children and adolescents, family socioeconomic status (*N* = 9 papers) and parental education (*N* = 7 papers) were found to have no association with their physical activity level. Age as a factor of physical activity showed inconsistent results, this may be attributed to the limited range in age of most studies, thus it was difficult to arrive at any conclusion. In adolescents, self-efficacy was also investigated (*N* = 4 papers) and showed a consistently positive association with physical activity. In addition, we found some factors that are not presented in the Table 2 due to insufficient papers for summarized results. For more information see Additional file 2.

## Discussion

For this review, we identified 42 papers published in English summarizing potential factors influencing physical activity in Chinese children and adolescents, and further identified 11 factors that were investigated in at least three studies. The results showed boys were consistently more active than girls. The parental physical activity was positively associated with children and adolescents’ physical activity. Children in suburban/rural regions showed less activity than in urban regions, and, specifically in adolescents, self-efficacy was positively associated with physical activity.

For the difference in physical activity between rural and urban regions, our finding is different from studies in western countries. A review showed no difference in children’s physical activity between rural and urban areas in the United States [69]; another study in Brazil showed children from rural areas were more active than those from urban areas. The direction and significance of rural-urban difference in physical activity might vary by the type of population, how long ago the study was done, and also by the method of physical activity measurement [70]. One study found children who lived in rural areas of China also showed an obviously increasing trend towards overweight or obesity prevalence in recent years [71], as physical activity is an acknowledged component of energy-balance-related behaviors. One explanation is that this might be due to the rapid industrialization and environmental contamination in the Chinese countryside, limited number of children’s playgrounds, and lack of physical activity facilities compared to urban schools. Another reason explaining this is that children, in rural areas especially, watch TV frequently, and longer TV-watching time may limit the chances for physical activity [72]. One study found that the daily viewing time of children in China’s rural areas significantly increased from 0.7 h to 1.7 h between 1997 and 2006 [73].

Families who live in urban areas usually have higher levels of education and socioeconomic status compared to suburban/rural areas, but there is only a small difference in family media equipment (television, mobile phones) between urban and rural areas, except for some western regions of China [74]. That might explain the reason that we found no association between parental education or family socioeconomic status and physical activity in this review. It should be noted however that the studies on urbanization, household income, and parental education were often of low quality. Future research is needed to find out whether this difference between rural/sub-rural and urban areas is related to environmental factors outside the household, such as the neighborhood characteristics or school environment.

The levels of physical activity included in this review were reported in various ways, but the low level of physical activity was clearly shown in most studies in Fig. 2, especially in Chinese adolescents. The result of age was inconclusive in Table 2, since most studies included a limited range of ages, and so it was difficult to arrive at any conclusion. A decrease in physical activity related to age during adolescence has been reported before in North American children [75, 76]. A recent systematic review of European children and adolescents also supported the same point [77]. Chinese adolescents (13–18 years) are in middle/high school, and under pressure from high school or college entrance examinations. Compared to children, adolescents spend more time in school and doing homework. Information on policy-influencing physical activity is scarce for Chinese children and adolescents, and further confirmation is needed, since a decrease in physical activity with increasing age and thus a change in energy balance may contribute substantially to the obesity epidemic. An effective public health policy to promote physical activity during school age may be of benefit to Chinese society [71, 78].

The finding that male sex was a consistently positive correlate in Chinese children and adolescents is in agreement with previous reviews [78, 79]. Apparently, boys report more physical activity than girls vis-à-vis different cultures and populations. The correlation between parental physical activity and children or adolescents physical activity was consistently positive in the Chinese studies. This was confirmed in some studies from different countries [80, 81], although not always consistently in the reviews [82, 83]. Chinese mothers have a long and deep influence on children’s lifestyles in traditional Chinese culture, much like mothers in other societies. This finding also suggests that to increase physical activity in children, parents need to be active themselves. Family-based interventions to increase physical activity level may be most effective if parents and children are encouraged to engage in physical activities together.

Self-efficacy for adolescent physical activity was defined as a young person’s belief in his/her ability to participate in physical activity and to perform physical activity despite existing barriers [84]. It has been identified as a determinant or mediator of physical activity in adolescents in previous reviews, mostly in developed countries [85, 86]. We identified that self-efficacy was consistently positively associated with adolescent physical activity from four papers of relatively high quality, of which two studies had a longitudinal design with self-efficacy measured by different scales, validated or adapted from previously validated measurements [87, 88]. This suggests that programs to promote physical activity in adolescents, which strengthen physical activity self-efficacy, have a high potential for being effective.

The adapted ecological model by the Lancet Physical Activity Series Working Group provides a comprehensive overview of the possible correlates of daily physical activity [89]. Based on the findings of Bauman and colleagues, combined with the findings from this review, we can conclude that, in particular, the psychological, cognitive, social, and environmental factors were difficult to summarize due to limited research. An understanding of the physical environmental correlates of transport and leisure-time activity in a developing country such as China is urgently needed to support the development of interventions to reverse the rapid trend towards inactivity. The inactivity trend may be driven by urbanization, passive entertainment, and motorized transport. For young people, this is especially important, because children and adolescents have less autonomy in their behaviors and are more likely than adults to be influenced by the environment, directly (through parents or peers) or indirectly [90, 91]. For example, active rather than passive videogames may stimulate physical activity in children [59], and increasing awareness of neighborhood sport facilities or building more such facilities may help active adolescents maintain or increase their leisure time PA [40, 51].

According to the results of our methodological quality assessment, lack of a longitudinal study design and using indirect measurements for outcome or determinants assessment have limited the quality of the research included in this review. To provide a full description of factors examined, we kept low quality papers in Table 2. Sensitivity analysis showed that the present results would not be changed wether low quality studies were excluded or not. High quality longitudinal studies using accelerometry or other objective devices to measure daily physical activity are needed to better understand the low level of activity, and how this relates to urbanization, fast economic development, and the rapidly changing living environment.

One limitation in this review is the limited number of studies of good quality. Another limitation is that only English-language published studies were included in our review. Papers published in Chinese might have given more information on this topic. A search till Dec, 10^th^, 2014 in Weipu, Wanfang and the CNKI Chinese Database resulted in 22 papers. However, the quality of these papers was low. Mostly papers published in Chinese were cross-sectional studies and used an invalid or poorly validated physical activity measurement tool. Moreover, for non-Chinese speakers, data of papers published in Chinese are not accessible for verification or reference. Furthermore, the majority of these studies were also published in internationally peer-reviewed journals in English. The inclusion criteria to publish in internationally peer reviewed journals in English guarantees a minimum in report quality as well as general accessibility to the results.

## Conclusion

The present review shows that Chinese children and adolescents have a low level of physical activity. Gender, urbanization, parental physical activity, and self-efficacy are important factors influencing physical activity. These factors could be taken into consideration in order to design effective interventions to counteract or halt the trend towards inactivity in young people. The results also suggest that the factors influencing the physical activity of Chinese children and adolescents are not yet fully understood, due to limited research quality and inconclusive findings. Future research is required to enhance our understanding of other influences, such as the physical environment, especially in early childhood.

## Additional files

Additional file 1: Criteria of Quality assessment. (DOCX 53 kb)
Additional file 2: Characteristics of the papers identified in this review. (DOCX 94 kb)
Additional file 3: Methodological quality assessment per quality item and per study. (DOCX 24 kb)

## Abbreviations

PRISMA: Preferred Reporting Items for Systematic Reviews and Meta-Analyses guidelines
QUIPS: Evaluation of the quality of prognosis studies in systematic reviews
STROBE: Strengthening The Reporting of OBservational studies in Epidemiology statement
