# Supplementary material for: Factors of physical activity among Chinese children and adolescents: a systematic review
Source: Int J Behav Nutr Phys Act. 2017 Mar 21;14:36. doi: 10.1186/s12966-017-0486-y (PMC5360041; doi:10.1186/s12966-017-0486-y)
Supplement: Additional file 1: — Criteria of Quality assessment. (DOCX 53 kb) [file 12966_2017_486_MOESM1_ESM.docx]

### Additional file1. Criteria of quality assessment

|  | Items to be considered for assessment of potential opportunity for bias |
| --- | --- |
| Study design | Was the study design a longitudinal study? |
| Study participation | Does the study sample represent the population of interest in terms of key characteristics?  The study should describe details of the participant eligibility criteria, sources, and methods of selection of participants. There should be adequate participation in a study by eligible individuals. Response rate should be adequate (at least 80%). |
| Outcome measurement |  |
|  | Did the study report a clear description of the outcome of physical activity, and did the instruments have acceptable quality?  Direct measurement by accelerometer (pedometer or heart rate monitor) and by questionnaire, which reported reliability or validity, or referred to any documentation on reliability or validity of the tools, were classified as of acceptable quality. |
|  |  |
|  |  |
|  |  |
| Related factors measurement |  |
|  | Did related factors measurement use validated methods and describe details of assessment?  A clear description of related factors measurement was provided. The factors measurement should be used as a valid tool; sociodemographic factors should be present as appropriate cut-off points. |
|  |  |
| Data analysis |  |
|  | Were the statistical tests used to assess the main outcomes appropriate?  There was sufficient presentation of data to assess the adequacy of the analysis. Important potential confounders should be included in the analysis, by stratification or modeling. |
