# Supplementary material for: Factors of physical activity among Chinese children and adolescents: a systematic review
Source: Int J Behav Nutr Phys Act. 2017 Mar 21;14:36. doi: 10.1186/s12966-017-0486-y (PMC5360041; doi:10.1186/s12966-017-0486-y)
Supplement: Additional file 2: — Characteristics of the papers identified in this review. (DOCX 94 kb) [file 12966_2017_486_MOESM2_ESM.docx]

Additional file 2. Characteristics of the papers identified in this review

| **Paper No.**  **(Authors & year)** | **Sample**  n (female %)  Age:(M±SD years)  Design & Geographical location | **Methods for assessing PA**  (reference list below) | **Level of PA**  **(**M±SD) | **Main findings** |
| --- | --- | --- | --- | --- |
| [25]  (Rowlands et al., 2002) | n=50(52)  Age: 8-11, (9.1±0.9)  Design: cross-sectional  Location: Hong Kong | CSA uniaxial accelerometer (Model 7164) [93] | Average daily count 7 days (counts/day) (299,384-140,427),  weekdays (299,407±147,075), weekend days, (311,732-173,137). | Physical activity negatively correlated with body fat (sum of skinfolds) in boys but not girls. There was no main effect for gender on physical activity. |
| [26]  (Cheng et al., 2003) | n=260(100)  Age: 11-18(14.7±1.5)  Design: cross-sectional  Location: Hong Kong | Questionnaire | Leisure time physical activity:  (2.62±4.58)MET hour/week.  Met PA recommendation: 19.2%. | Physical activity participation correlated with perceived benefits (body image and health) in female adolescents. |
| [27]  (Tudor-Locke et al., 2003) | n=2675(46.8)  Age: 6-18 (11.5±3.3)  Design: cross-sectional  Location: Eight provinces in mainland China: Guangxi, Guizhou, Heilongjiang, Henan, Hubei, Hunan, Jiangsu and Shandong. | Questionnaire of the China Health and Nutrition Survey [94] | 72% of children and adolescents engage in in-school MVPA for a median of 90-110 min./week. Relatively few children and adolescents (8%) participate in any MVPA outside of school. | Female (≥12 yrs.) engaged in shorter durations of MVPA during school hours compared to males. |
| [28]  (Liou and Chiang, 2004) | n=463(46.2)  Age: 9-12(10.8)  Design: cross-sectional  Location: Taiwan | Modified version of the “Three-day Physical Activity Logs (3-d PAL)” [95] | MVPA (min./day)  boys: (152.4±113.5),  girls: (160.7±113.6).  Met PA recommendation: 79.4%. | There were no significant main effects for age or gender, and no interaction was found between energy expenditure and MVPA. |
| [29]  (Wu and Pender, 2005) | n=583(48.7)  Age: 13-16(14.7±0.6)  Design: longitudinal (one year follow-up)  Location: Taiwan | Modified version of the Child/Adolescent Activity Log-CAAL  [96] | No report | Gender, social support, modeling, self-efficacy, and perceived benefits and barriers to performing physical activity influence physical activity in adolescents directly and indirectly. Adolescents with higher parent education levels engaged in less physical activity. |
| [30]  (Shi et al., 2006) | n=824(47.7)  Age: 12-14  Design: cross-sectional  Location: Zhenjiang and Xuzhou (Jiangsu province) | Questionnaire | 45.2 % of adolescents had vigorous physical activity≥3 times/week, 38.5% engaged in low level PA, while 26.1% engaged in high level PA (Physical activity level was based on three variables: active commuting to school, housework, and vigorous physical activity). | Girls in rural areas had a high physical activity score. High household socioeconomic status and low education of father were associated with low physical activity among boys. Age and region (south/north) were not significant. |
| [31]  (Chen et al., 2007) | n=2235(48.2)  Age: 12-18  Design: cross-sectional  Location: Taiwan | Personal Health Behaviors and Adolescent Questionnaire [97] | The prevalence of engaging in any level of physical activity was 78.2% among adolescents.  Met PA recommendation: 28.4%. | Boys and urban adolescents were more active than girls and rural adolescents. The prevalence of physical activity declined with age. Other factors associated with physical activity included educational status, smoking, and weight control. Parental education and weight status were not associated with physical activity. |
| [32]  (Li et al., 2007) | n=1760(50)  Age: 11-17  Design: cross-sectional  Location: Xian (Shanxi province) | Modified version of “Adolescents Physical Activity Recall Questionnaire” [98] | Light physical activity: boys, 1.4 hours/week, girls, 1.1 hours/week; Moderate physical activity: boys, 8.6 hours/week, girls, 7.4 hours/week; Vigorous physical activity: boys, 4.7 hours/week, girls, 2.3 hours/week.  Met PA recommendation: 56%. | Males spent more hours in light and vigorous activities than females. Those living in rural areas spent significantly more hours doing light physical activities. There were no differences in the activity percentage (by guidelines) for age and wealth index. |
| [33]  (Chen et al., 2008) | n=331(52)  Age: 7-8  Design: cross-sectional  Location: Taiwan | Children’s self-administered physical activity checklist (SAPAC) [99] | No report | More moderate and vigorous activity METs (MVPAMETs) were related to less authoritarian parenting in boys and higher skinfold thickness in girls.  Attending an urban school was found to contribute to the variance in more MVPAMETs in girls. |
| [34]  (Li et al., 2009) | n=2500  Age: 6-18  Design: cross-sectional  Location: Nine provinces in mainland China (Guangxi, Guizhou, Heilongjiang, Henan, Hubei, Hunan, Jiangsu, Liaoning, and Shandong). | Questionnaire of the China Health and Nutrition Survey [100] | 52.8% of children spent more than 2 hours in physical activities per week. | Urban children reported more time exercising than rural children. Children’s exercise time was associated with fathers’ exercise time, but not associated with mothers’ exercise time. |
| [35]  (Wang et al., 2009) | n=91(49.5)  Age: 11-14  Design: cross-sectional  Location: Beijing | HR monitor (RS 400, Polar Electro, Kempele, Finland) & questionnaire | Net HR at leisure time (beats/min):  boys (22.57±4.20),  girls (22.86±4.58). | Severe iron deficiency impairs habitual physical activity. |
| [36]  (Huang et al., 2010) | n=523(51.5)  Age: 11-12  Design: cross-sectional  Location: Taiwan | Modified version of the Child/Adolescent Activity Log-CAAL[4]  & adapted short form International Physical Activity Questionnaire (IPAQ) | Energy consumption  (MET .hrs. per week):  urban children (89±56.54),  rural children (78.25±52.99). | Multiple regression results showed gender and accessibility to facilities had a significant impact on the children’s physical activity. Geographical location in either rural or urban areas, educational level of the head of household, monthly family income, and walkability had no correlation with physical activity. |
| [37]  (Lam et al., 2010) | n=1147(46.7)  Age: 9-13(10.45)  Design: cross-sectional  Location: Hong Kong | Adapted Questionnaire of CHNS [101] | MVPA (min/week)  boys: 389±371;  girls: 375±353. | Older children spent more time in MVPA, and there was no difference in MVPA between boys and girls. |
| [38]  (Luszczynska et al., 2010) | n=534(54)  Age: 12-18(13.8±1.4)  Design: longitudinal (four weeks follow-up)  Location: Central region of China | Questionnaire | Index of leisure time physical activity,  Time 1: 8.82±4.59  Time 2: 10.41±6.46 | Physical activity was predicted by baseline activity, planning, self-efficacy, and the self-efficacy*planning interaction. |
| [39]  (Pang and Ha, 2010) | n=335(48.7)  Age: 10-11(10.71)  Design: cross-sectional  Location: Hong Kong | Modified Chinese version of Physical Activity Questionnaire for Children (PAQ-C) [102] | Physical activity participation (MVPA): 2.24±0.54. | Children’s perceived subjective task value (attainment, intrinsic, and utility value) correlated with their physical activity participation. |
| [40]  (Wong et al., 2010) | n=29,139(56.5)  Age: (14.5±0.13)  Design: cross-sectional  Location: Hong Kong | Questionnaire | Met PA recommendation: 10.2%. | Perceived availability of sports facilities was positively associated, and that of computer/Internet negatively associated with being sufficiently active. |
| [41]  (Xu et al., 2010) | n=2375(53.8)  Age: 13-15(13.9±1.0)  Design: cross-sectional  Location: Nanjing (Jiangsu province) | Adapted Chinese version of IPAQ [103] | Recreational physical activity time: 11.6±5.3 (hrs./wk.) | Boys were more likely to spend more time on physical activity. Residential density was negatively associated with recreational physical activity time. |
| [42]  (Dearth-Wesley et al., 2012) | n=353(46.7)  Age: (6-9 at baseline, <12 throughout the study)  Design: longitudinal (two to four years follow-up)  Location: Nine provinces in mainland China (Guangxi, Guizhou, Heilongjiang, Henan, Hubei, Hunan, Jiangsu, Liaoning, and Shandong). | Questionnaire of the China Health and Nutrition Survey [27] | Leisure-time MVPA (MET .hrs. per week):  2000 Cohort: 7.5 at baseline, and 21.1 at follow-up.  2004 Cohort: 10.6 at baseline, and 14.7 at follow-up.  Met PA recommendation: 15% on average. | Children experienced increases in leisure-time sports activities with increasing age. Mother-child associations were positive for leisure-time sports activities. Household location (urban/rural), household income, and maternal education were not associated with children’s activities. |
| [43]  (Guo et al., 2012) | n=4445(48.3)  Age: 5-18  Design: cross-sectional  Location: Liaoning province | Questionnaire | Met PA recommendation: 55.5%. | Boys were more active than girls. A family income ranging from RMB 2000 to RMB 5000 per month was associated with a higher possibility of having appropriate physical activity than an income less than RMB 2000. Parental education level had no influence on physical activity. |
| [44]  (Cheung, 2012) | n=456(49.6)  Age: 10-12(11.4±0.97)  Design: cross-sectional  Location: Hong Kong | Three-Day Physical Activity Recall (3DPAR) [104] | Children spent the most time in light intensity PA (6.07 time blocks) and the least time in higher intensity PA (moderate =1.19; hard=0.53; very hard=0.11) | Boys spent more time blocks on high intensity PA than girls. Children who participated in organized PA programs spent fewer time blocks on light intensity PA and more time blocks in vigorous PA. |
| [45]  (Wen and Hui, 2012) | n=1869(48.6)  Age: 10-15(12.5±0.9)  Design: cross-sectional  Location: Shantou (Guangdong province) & Ganzhou (Jiangxi province) | Physical activity rating questionnaire for children and youth (PARCY) [105] | PA level score: (5.1±2.8) | Parents’ responsiveness, demandingness, and diet- and physical-activity monitoring were found to be related to adolescents’ physical activity. |
| [46]  (Cao et al., 2013) | n=534(52)  Age: 13.95±1.67  Design: longitudinal (four weeks follow-up)  Location: Central region of China | Adapted 7-day physical activity recall questionnaire (IPAQ) [106] | Leisure time physical activity (minutes/week):  Time 1: 85.48 ±84.53,  Time 2: 86.77± 74.35. | Adolescents who had more skills were more likely to successfully translate their intentions into plans. (Prospective anticipation of when, where, and how to  perform physical activities.) |
| [47]  (Huang et al., 2013) | n=280(52.1)  Age: 11.1±0.9  Design: cross-sectional  Location: Hong Kong | Children’s Leisure Activities Study Survey questionnaire- Chinese version (CLASS-C) [107] | MVPA: (min./day):  boys, 72.0±50.6,  girls, 75.9±52.7.  Met PA recommendation: 52.9%. | Participation in school sports teams and self-efficacy were positively associated with PA in boys. Girls who reported participation in school sports teams, who perceived more peer support, had a more supportive home PA environment, and those who spent more time doing homework were more physically active. |
| [48]  (Wang et al., 2013) | n=2163(49.8)  Age: 9-17(13.41±2.25)  Design: cross-sectional  Location: Eleven cities in China (Chengdu, Fuyang, Ganzhou, Guangzhou, Shanghai, Shenyang, Tianjin, Tongzhou, Wenzhou, Xian and Yingtan) | Accelerometers (Actigraph GT3X or GT3X+) | Chinese-specific cutoff points:  MVPA(min./day):28.26±17.66.  Met PA recommendation:  9-12 years old (3.6%), 13-17 years old (7.2%), total (5.6%).  Freedson’s cut-off points: MVPA(min/day):76.94±37.02.  Met PA recommendation:  9-12 years old (81.5%), 13-17 years old (45.5%), total (61.1%). | Chinese children and youth were more active during weekdays than during weekend days, and boys were more active than girls. No difference in physical activity was found across different BMI categories. |
| [49]  (Zhang et al., 2013) | n=19,523(49.9)  Age: 13-18  Design: cross-sectional  Location: Shandong province | Questionnaire | Met PA recommendation: 29.1%. | Weight status: overweight/obese adolescents had poor PA status compared with underweight/normal weight adolescents. |
| [50]  (Li et al., 2014) | n=497(48.3)  Age: 8-10  Design: cross-sectional  Location: Guangzhou (Guangdong province) and Hechi (Jiangxi province) | Godin Leisure-time Exercise Questionnaire [108] | Met PA recommendation: 84.9%. | Children whose mothers reported engaging in exercise sometimes or frequently were over four times as likely to meet the daily recommended level of MVPA, compared to children whose mothers never or rarely exercised. Children who lived with one grandparent were also more likely to achieve at least 60 min. of MVPA per day, compared with children who lived away from all grandparents. |
| [51]  (Wong et al., 2014) | n= 9993(63.2)  Age: 14±1.7  Design: longitudinal (16 months follow-up)  Location: Hong Kong | Questionnaire | Weekly frequency of leisure time MVPA for at least 30 minutes.  Subjects number (%).  None: baseline, n=2179(21.8); follow-up, n=2418(24.2).  Less than once a week: baseline, n=2728(27.3); follow-up, n=2558(25.6).  1-3 times a week: baseline, n=3298(33.0); follow-up, n=3708(37.1).  4-6 times a week: baseline, n=819(8.2); follow-up, n=560(5.6).  Daily or more: baseline, n=969(9.7); follow-up, n=749(7.5). | Increasing awareness of neighborhood sport facilities or building more such facilities may help active adolescents maintain or increase their leisure time PA. |
| [52]  (Duan et al., 2015) | n=1715(48.5)  Age: 12-15 (13.3±1.0)  Design: cross-sectional  Location: Beijing | Adapted Questionnaire of CHNS [27] | Met PA recommendation: 53.7%. | Boys spent more time engaged in physical activity than girls did. Each 10-unit increase in attitudes toward physical education was associated with increased odds of 1.15 for spending more than 1 hr./day on MVPA. Students in suburban schools reported engaging in physical activity less, when compared with those in urban schools. |
| [53]  (Gao et al., 2015) | n=68(58.8)  Age: 10-11  Design: cross-sectional  Location: Hong Kong | Pedometers (SW 700,YAMAX Corporation, Tokyo, Japan) | Total daily steps: 9341±2478. | Boys were more active than girls. Students accumulated 914 steps more on days that included PE classes than on days without PE classes. No significant differences in body weight status (normal weight vs. overweight and obesity) and travel mode (active vs. passive modes). |
| [54]  (Ho et al., 2015) | n=775(55.8)  Age: 12-14(12.28±0.77)  Design: cross-sectional  Location: Hong Kong | Physical Activity Rating Questionnaire for Children and Youth (PARCY) [105] | Physical activity level score: 5.41, corresponding to light daily PA for at least 20 minutes. | Physical activity level correlated with the adolescent’s mental well-being, self-efficacy, resilience, and socioeconomic status, but not with age, school connectedness, or family connectedness. |
| [55]  (Wang et al., 2015) | n=7286(47.7)  Age: 13-15  Design: cross-sectional  Location: Hangzhou (Jiangsu province) | Questionnaire | Met PA recommendation: 9%. | Families’ involvement in their adolescents’ physical activity on most days of the week was associated with a higher level of adolescents’ MVPA. |
| [56]  (Wong et al., 2015) | n= 412(46.0)  Age: Baseline, 7.8±1.0;  1 year follow up, 8.6±1.0;  2 year follow up, 9.5±1.0.  Design: longitudinal  Location: Hong Kong | Acti-Graph GT3X accelerometer (ActiGraph, Pensacola, Florida, USA) | MVPA (min/d):  baseline, 61.5±23.3;  1 year follow up, 50.0±20.4;  2 year follow up, 45.6±20.7. | Chinese children were more physically active on weekends than weekdays. An age-related decline in MVPA was more marked on weekends than weekdays. |
| [57]  (Dong et al., 2016) | n=5201  Age: 7-17  Survey 1991, 12.6±3.2(48.6),  Survey 2000, 13.0±2.9(47.3),  Survey 2009, 14.2±2.3(46.8).  Design: longitudinal  Location: Nine provinces in mainland China (Guangxi, Guizhou, Heilongjiang, Henan, Hubei, Hunan, Jiangsu, Liaoning, and Shandong). | Questionnaire | No report | Children were more likely to participate in leisure-time sports if their parents participated in leisure-time sports. |
| [58]  (Huang et al., 2016) | n= 672(46.6)  Age: 7.6±1.0  Design: longitudinal (2 year follow up)  Location: Hong Kong | ActiGraph GT3X+ accelerometer | No report | Low correlations were observed between different behavioral variables and MVPA. LPA and sleep was positive associated with MVPA, screen time and academic-related activities were negative associated with MVPA. |
| [59]  (Lau et al., 2016) | n= 80(31.3)  Age: 8-11 (9.23±0.52)  Design: longitudinal (12 weeks follow-up)  Location: Hong Kong | ActiGraph GT3X+ accelerometer. | MVPA (min/d):  Intervention group, baseline, 19.20±7.50, Post-test, 29.33±14.67; Control group, baseline, 20.36±8.50, Post-test, 23.71±12.40. | A 12-week (60 minutes twice per week) school-based Active video games (AVGs) intervention can improve Chinese children’s PA level. These findings indicated that AVGs could be used as an alternative means to engage Chinese children in PA in school setting. |
| [60]  (Wang JJ et al., 2016) | n= 449(43.9)  Age: 8-13 (10.2±1.1)  Design: cross-sectional  Location: Hong Kong | ActiGraph GT3X accelerometers, Physical Activity Questionnaire for Older Children (PAQ-C) [109] | PAQ-C Score: 2.71±0.70; Objective MVPA (min/day): 43.09±12.74. | Higher levels of self-efficacy and autonomous motivation were positively related to PA level. |
| [61]  (Wang L & Qi J, 2016) | n= 612(48.1)  Age:10-16(12±1.2)  Design: cross-sectional  Location: Shanghai | Actigraph GT3X accelerometers [110] | The participating children spent an average of (22±14.5) minutes/day in MVPA based on the Chinese specific cutoff points.  Met PA recommendation: 4.7%. | Adolescents living in single-parent households and step families were more physically active than those living in two-parent homes and with biological parents, respectively. However, adolescents residing with grandparents were less active than those living with neither grandparent. |
| [62]  (Wang L & Zhang Y, 2016) | n= 488 (42.8)  Age:12-16 (13.91±0.96)  Design: cross-sectional  Location: Shanghai | Godin Leisure Time Exercise Questionnaire  [111] | No report | Self-efficacy was the only significant predictor of physical activity behaviour(MVPA) in the multiple regression model, when all variables were entered into the equation including intention, past physical activity behaviour and perceived behaviour control. |
| [63]  (Wang X et al., 2016) | n= 1032(47.8)  Age:12-15, boys (13.8±0.8), gilrs (13.9±0.9).  Design: cross-sectional  Location: Xi an | Physical Activity Questionnaire for Middle School Students (PAQMSS) [112] | MVPA (min/week): Median (25th-75th), Boys, 610 (290-1215); Girls, 390 (205-775).  Met PA recommendation: 63.4%. | Obesity and high socioeconomic status (SES) were positively associated with IPA, whereas male sex, underweight status, and high family support level for physical activity showed inverse associations. Age was not associated with IPA. |
| [64]  (Xu et al., 2016) | n= 1208  Age: 3-7 (5±0.8)  Design: cross-sectional  Location: Chongqing, Chengdu, Taiyuan, and Shijiazhuang | Questionnaire | Minutes spend on PA throughout the day: Overweight and Obese (113.27±72.91); Normal Weight (101.25±53.25); Underweight (95.04±50.70). | Positive correlations were found between maternal social cognition and preschool children’s physical activity (PA) behavior. |
| [65]  (Yeung et al., 2016) | n= 2517 (49.0)  Age: 13-19(15.65±1.71)  Design: cross-sectional  Location: Hong Kong | International Physical Activity Questionnaire-Short Form (IPAQ-SF) [113] | MVPA(min/wk): 121.32±210.80 | Children's intention to participate in PA was a strong predictor of their engagement in MVPA. Parents' exercise habit had both direct and indirect (via attitude) effects on their children's intention to participate in PA. Children's attitude toward PA, parents' exercise habit, and SES had significant effects on the children's intention to participate in PA. |
| [66]  (Zheng et al., 2016) | n= 770(48.3)  Age: boys, 9.3±1.8;  girls, 9 .4±1.7.  Design: cross-sectional  Location: seven urban areas (Beijing, Guangzhou, Chengdu, Shenyang, Suzhou, Lanzhou, and Zhengzhou) and two rural areas (a lowland area and a mountainous area, both in Hebei province) | Questionnaire | Leisure-time PA: ≥60 mins/week, boys, n=254(63.8%); girls, n=266(71.5%);  School-time PA: ≥100 mins/week, boys, n=276(69.3%); girls, n=235(63.2%). | Physical activity was significantly associated with dyslipidemia in both genders. Increasing leisure-time physical activity for boys and school-time physical activity for girls may be critical. |

**References**

[93] Eston RG, Rowlands AV, Ingledew DK. Validity of heart rate, pedometry, and accelerometry for predicting the energy cost of children's activities. J Appl Physiol (1985). 1998;84(1):362-71.

[94] China Health and Nutrition Survey. Questionnaires: 1997 survey. Available at: http://www.cpc.unc.edu/projects/china/data/questionnaires. Accessed Feb/15, 2016.

[95] Bouchard C, Tremblay A, Leblanc C, Lortie G, Savard R, Theriault G. A method to assess energy expenditure in children and adults. Am J Clin Nutr. 1983;37(3):461-7.

[96] Garcia AW, George TR, Coviak C, Antonakos C, Pender NJ. Development of the child/adolescent activity log: a comprehensive and feasible measure of leisure-time physical activity. Int J Behav Med. 1997;4(4):323-38; doi:10.1207/s15327558ijbm0404_5.

[97] Lan TY, Chang HY, Tai TY. Relationship between components of leisure physical activity and mortality in Taiwanese older adults. Prev Med. 2006;43(1):36-41; doi:10.1016/j.ypmed.2006.03.016.

[98] Booth ML, Okely AD, Chey TN, Bauman A. The reliability and validity of the Adolescent Physical Activity Recall Questionnaire. Med Sci Sports Exerc. 2002;34(12):1986-95; doi:10.1249/01.MSS.0000038981.35052.D3.

[99] Sallis JF, Strikmiller PK, Harsha DW, Feldman HA, Ehlinger S, Stone EJ, et al. Validation of interviewer- and self-administered physical activity checklists for fifth grade students. Med Sci Sports Exerc. 1996;28(7):840-51.

[100] China Health and Nutrition Survey. Questionnaire: 2000 survey. Available at: http://www.cpc.unc.edu/projects/china/data/questionnaires. Accessed Feb/15, 2016.

[101] China Health and Nutrition Survey. 2006 Child questionnaire. Available at: http://www.cpc.unc.edu/projects/china/data/questionnaires. Accessed Feb/15, 2016.

[102] Crocker PR, Bailey DA, Faulkner RA, Kowalski KC, McGrath R. Measuring general levels of physical activity: preliminary evidence for the Physical Activity Questionnaire for Older Children. Med Sci Sports Exerc. 1997;29(10):1344-9.

[103] Qu NN, Li KJ. Study on the reliability and validity of international physical activity questionnaire (Chinese Vision, IPAQ). Zhonghua Liu Xing Bing Xue Za Zhi. 2004;25(3):265-8.

[104] Lee KS, Trost SG. Validity and reliability of the 3-day physical activity recall in Singaporean adolescents. Res Q Exerc Sport. 2005;76(1):101-6; doi:10.1080/02701367.2005.10599265.

[105] Criterion-related validity of a 0–10 scale physical activity rating in Chinese youth. Proceedings of the 2001 Asia-Pacific Rim Conference on Exercise and Sports Science: the new perspective of exercise & sports science for the better life in the 21st century.; July; Seoul, Korea: Seoul National University.; 2001.

[106] Craig CL, Marshall AL, Sjostrom M, Bauman AE, Booth ML, Ainsworth BE, et al. International physical activity questionnaire: 12-country reliability and validity. Med Sci Sports Exerc. 2003;35(8):1381-95; doi:10.1249/01.MSS.0000078924.61453.FB.

[107] Huang YJ, Wong SH, Salmon J. Reliability and validity of the modified Chinese version of the Children's Leisure Activities Study Survey (CLASS) questionnaire in assessing physical activity among Hong Kong children. Pediatr Exerc Sci. 2009;21(3):339-53.

[108] Godin G, Shephard R. Godin leisure-time exercise questionnaire. Med Sci Sports Exerc. 1997;29(6):36-8.

[109] Wang JJ, Baranowski T, Lau WP, Chen TA, Pitkethly AJ. Validation of the Physical Activity Questionnaire for Older Children (PAQ-C) among Chinese Children. Biomed Environ Sci. 2016;29(3):177-86; doi:10.3967/bes2016.022.

[110] Janz KF. Validation of the CSA accelerometer for assessing children's physical activity. Med Sci Sports Exerc. 1994;26(3):369-75.

[111] Godin G, Shephard RJ. A simple method to assess exercise behavior in the community. Can J Appl Sport Sci. 1985;10(3):141-6.

[112] Fogelholm M, Malmberg J, Suni J, Santtila M, Kyrolainen H, Mantysaari M, et al. International Physical Activity Questionnaire: Validity against fitness. Med Sci Sports Exerc. 2006;38(4):753-60; doi:10.1249/01.mss.0000194075.16960.20.

[113] Macfarlane DJ, Lee CC, Ho EY, Chan KL, Chan DT. Reliability and validity of the Chinese version of IPAQ (short, last 7 days). J Sci Med Sport. 2007;10(1):45-51; doi:S1440-2440(06)00086-7.
