# Supplementary material for: Factors of physical activity among Chinese children and adolescents: a systematic review
Source: Int J Behav Nutr Phys Act. 2017 Mar 21;14:36. doi: 10.1186/s12966-017-0486-y (PMC5360041; doi:10.1186/s12966-017-0486-y)
Supplement: Additional file 3: — Methodological quality assessment per quality item and per study. (DOCX 24 kb) [file 12966_2017_486_MOESM3_ESM.docx]

Additional file 3. Methodological quality assessment per quality item and per study

| No. | Authors. year | Study design | Study  participation | Outcome  measurement | Related factors  measurement | Data analysis | Total |
| --- | --- | --- | --- | --- | --- | --- | --- |
| [25] | Rowlands et al. 2002 | 0 | 0 | 1 | 1 | 1 | 3 |
| [26] | Cheng et al. 2003 | 0 | 1 | 1 | 1 | 0 | 3 |
| [27] | Tudor-Locke et al. 2003 | 0 | 0 | 1 | 1 | 1 | 3 |
| [28] | Liou and Chiang. 2004 | 0 | 1 | 1 | 1 | 1 | 4 |
| [29] | Wu and Pender. 2005 | 1 | 0 | 1 | 1 | 1 | 4 |
| [30] | Shi et al. 2006 | 0 | 1 | 0 | 1 | 0 | 2 |
| [31] | Chen et al. 2007 | 0 | 1 | 1 | 1 | 1 | 4 |
| [32] | Li et al. 2007 | 0 | 1 | 1 | 1 | 0 | 3 |
| [33] | Chen et al. 2008 | 0 | 0 | 1 | 1 | 1 | 3 |
| [34] | Li et al. 2009 | 0 | 1 | 1 | 1 | 0 | 3 |
| [35] | Wang et al. 2009 | 0 | 0 | 1 | 1 | 0 | 2 |
| [36] | Huang et al. 2010 | 0 | 0 | 1 | 1 | 1 | 3 |
| [37] | Lam et al. 2010 | 0 | 0 | 1 | 1 | 0 | 2 |
| [38] | Luszczynska et al. 2010 | 1 | 0 | 0 | 1 | 1 | 3 |
| [39] | Pang and Ha. 2010 | 0 | 1 | 1 | 1 | 1 | 4 |
| [40] | Wong et al. 2010 | 0 | 1 | 1 | 1 | 1 | 4 |
| [41] | Xu et al. 2010 | 0 | 1 | 1 | 1 | 1 | 4 |
| [42] | Dearth-Wesley et al. 2012 | 1 | 0 | 1 | 1 | 1 | 4 |
| [43] | Guo et al. 2012 | 0 | 1 | 0 | 0 | 1 | 2 |
| [44] | Cheung. 2012 | 0 | 1 | 1 | 1 | 0 | 3 |
| [45] | Wen and Hui. 2012 | 0 | 1 | 1 | 1 | 1 | 4 |
| [46] | Cao et al. 2013 | 1 | 0 | 1 | 1 | 1 | 4 |
| [47] | Huang et al. 2013 | 0 | 0 | 1 | 1 | 1 | 3 |
| [48] | Wang et al. 2013 | 0 | 0 | 1 | 1 | 1 | 3 |
| [49] | Zhang et al. 2013 | 0 | 1 | 0 | 1 | 1 | 3 |
| [50] | Li et al. 2014 | 0 | 1 | 1 | 1 | 1 | 4 |
| [51] | Wong et al. 2014 | 1 | 0 | 1 | 1 | 1 | 4 |
| [52] | Duan et al. 2015 | 0 | 1 | 1 | 1 | 0 | 3 |
| [53] | Gao et al. 2015 | 0 | 1 | 1 | 1 | 0 | 3 |
| [54] | Ho et al. 2015 | 0 | 1 | 1 | 1 | 1 | 4 |
| [55] | Wang et al. 2015 | 0 | 1 | 0 | 1 | 1 | 3 |
| [56] | Wong et al. 2015 | 1 | 0 | 1 | 1 | 1 | 4 |
| [57] | Dong et al. 2016 | 1 | 1 | 1 | 1 | 1 | 5 |
| [58] | Huang et al. 2016 | 1 | 0 | 1 | 1 | 1 | 4 |
| [59] | Lau et al. 2016 | 1 | 1 | 1 | 1 | 1 | 5 |
| [60] | Wang JJ et al. 2016 | 0 | 0 | 1 | 1 | 1 | 3 |
| [61] | Wang and Qi. 2016 | 0 | 0 | 1 | 1 | 1 | 3 |
| [62] | Wang and Zhang. 2016 | 0 | 0 | 1 | 1 | 1 | 3 |
| [63] | Wang X et al. 2016 | 0 | 1 | 1 | 1 | 1 | 4 |
| [64] | Xu et al. 2016 | 0 | 1 | 0 | 1 | 0 | 2 |
| [65] | Yeung et al. 2016 | 0 | 1 | 1 | 1 | 1 | 4 |
| [66] | Zheng et al. 2016 | 0 | 1 | 0 | 1 | 1 | 3 |

*A score was assigned to each study based on whether quality assessment items were met the criterion (score=1) or not (score=0). The scores were summed and described as low quality (0-2) or acceptable quality (3-5).
